# Abstract Representations of Emotions Perceived From the Face, Body, and Whole-Person Expressions in the Left Postcentral Gyrus

**DOI:** 10.3389/fnhum.2018.00419

**Published:** 2018-10-18

**Authors:** Linjing Cao, Junhai Xu, Xiaoli Yang, Xianglin Li, Baolin Liu

**Affiliations:** ^1^School of Computer Science and Technology, Tianjin Key Laboratory of Cognitive Computing and Application, Tianjin University, Tianjin, China; ^2^Medical Imaging Research Institute, Binzhou Medical University, Yantai, China; ^3^State Key Laboratory of Intelligent Technology and Systems, Tsinghua National Laboratory for Information Science and Technology, Tsinghua University, Beijing, China

**Keywords:** emotion, representational similarity analysis, fMRI, abstract representation, whole-brain searchlight

## Abstract

Emotions can be perceived through the face, body, and whole-person, while previous studies on the abstract representations of emotions only focused on the emotions of the face and body. It remains unclear whether emotions can be represented at an abstract level regardless of all three sensory cues in specific brain regions. In this study, we used the representational similarity analysis (RSA) to explore the hypothesis that the emotion category is independent of all three stimulus types and can be decoded based on the activity patterns elicited by different emotions. Functional magnetic resonance imaging (fMRI) data were collected when participants classified emotions (angry, fearful, and happy) expressed by videos of faces, bodies, and whole-persons. An abstract emotion model was defined to estimate the neural representational structure in the whole-brain RSA, which assumed that the neural patterns were significantly correlated in within-emotion conditions ignoring the stimulus types but uncorrelated in between-emotion conditions. A neural representational dissimilarity matrix (RDM) for each voxel was then compared to the abstract emotion model to examine whether specific clusters could identify the abstract representation of emotions that generalized across stimulus types. The significantly positive correlations between neural RDMs and models suggested that the abstract representation of emotions could be successfully captured by the representational space of specific clusters. The whole-brain RSA revealed an emotion-specific but stimulus category-independent neural representation in the left postcentral gyrus, left inferior parietal lobe (IPL) and right superior temporal sulcus (STS). Further cluster-based MVPA revealed that only the left postcentral gyrus could successfully distinguish three types of emotions for the two stimulus type pairs (face-body and body-whole person) and happy versus angry/fearful, which could be considered as positive versus negative for three stimulus type pairs, when the cross-modal classification analysis was performed. Our study suggested that abstract representations of three emotions (angry, fearful, and happy) could extend from the face and body stimuli to whole-person stimuli and the findings of this study provide support for abstract representations of emotions in the left postcentral gyrus.

## Introduction

The ability to understand the feelings of other people is part of successful social interactions in our daily life. Emotions can be perceived from various sensory cues, such as facial expressions, hand gestures, body movements, emotional whole-persons and vocal intonations (Gelder et al., 2006; Heberlein and Atkinson, 2009). These different sensory cues could elicit very similar emotions suggesting that the brain hosts “supramodal” or abstract representations of emotions regardless of the sensory cues. For example, fear can be recognized similarly from the eye region of faces, or postures and movements of body parts, suggesting that emotions might be represented at an abstract level. Numerous considerable efforts have been devoted to identify this kind of abstract representations that are invariant to the sensory cues (Peelen et al., 2010; Aube et al., 2015; Kim et al., 2015, 2017). Previous studies suggested that the medial prefrontal cortex (MPFC) contained representations of emotions that were invariant to perceptual modality (Peelen et al., 2010; Chikazoe et al., 2014) and generalized to emotions inferred in the absence of any overt display (Skerry and Saxe, 2014). And the neural representations in the MPFC and left superior temporal sulcus (STS) have been suggested to be modality-independent but emotion-specific (Peelen et al., 2010). By examining the neural representations of categorical valence (positive, neutral, and negative) elicited by visual and auditory modalities, modality-general representations were discovered in some specific regions, including the precuneus, bilateral MPFC, left STS/postcentral gyrus, right STS/middle frontal gyrus (MFG), inferior parietal lobe (IPL), and thalamus (Kim et al., 2017). Moreover, emotions were demonstrated to be indeed represented at an abstract level and the abstract representations could also be activated by the memories of an emotional event (Kim et al., 2015).

Although it has been demonstrated that facial and bodily emotions can be represented at an abstract level regardless of the sensory cue in specific brain regions (Peelen et al., 2010; Klasen et al., 2011; Chikazoe et al., 2014; Skerry and Saxe, 2014; Aube et al., 2015; Kim et al., 2017; Schirmer and Adolphs, 2017), the abstract representation is only elicited using one single face or body parts for the visual cue, suggesting that emotions could be similarly perceived by emotional faces or bodies. However, behavioral studies have suggested that the human brain can encode the whole-person expressions in a holistic rather than part-based manner (Soria Bauser and Suchan, 2013). Neuroimaging studies have also shown that body-selective areas preferred the whole-person to the sum of their parts (McKone et al., 2001; Maurer et al., 2002; Zhang et al., 2012). Another recent study found a preference of the whole-body to the sums of their scrambled parts in some body-sensitive areas (Brandman and Yovel, 2016), indicating a holistic representation of the whole-person expression. Therefore, the emotions of whole-person expressions should be explored individually rather than in an integrated way from the isolated emotional faces and bodies. Further, one of our latest study has found that in the extrastriate body area (EBA), the whole-person patterns were almost equally associated with weighted sums of face and body patterns, using different weights for happy expressions but equal weights for angry and fearful ones (Yang et al., 2018). So, it remains unclear how the whole-person’s emotion is represented in the human brain and whether the representations of emotions of the face, body, and whole-person expressions can be abstractly formed in specific brain regions.

A series of previous neuroimaging studies have utilized traditional univariate analyses to explore the cognitive mechanism of emotions, such as the general linear model (GLM). The GLM is voxel-based by estimating the activation of each voxel from specific experimental conditions, and only the statistically significant voxels were reported, which led to the loss of the fine-grained pattern information (Haynes and Rees, 2006; Norman et al., 2006). At present, advanced approaches such as multivoxel pattern analysis (MVPA) or representational similarity analysis (RSA) (Nikolaus et al., 2008) allows us to decode the pattern information across the whole brain. As compared with the multivariate decoding method that extracted features from multidimensional space and resorted to categorical judgment, RSA can provide us richer information on neural representations, which provides a framework for characterizing representational structure and for testing computational models of that structure (Hajcak et al., 2007; Kriegeskorte and Kievit, 2013). And it decodes neural information from the perspective of multivariate patterns and bridges the gap between different regions, subjects and species. In RSA, neural activity patterns can be abstracted from specific brain regions and then the dissimilarities of neural activity patterns elicited by different stimuli or conditions are computed. The representational dissimilarity matrices (RDMs) of the conditions characterize the information carried by a given representation in the brain. The neural RDMs can then be compared to the dissimilarity space captured by a specific model to test whether specific brain regions could match the representation of the model successfully. The significant correlations between neural RDMs and models suggested that the model could decode neural information of specific brain regions. And RSA has also been used to go beyond classification to test specific alternative models of the dimensions that structure the representation of others’ emotions, indicating that our knowledge of others’ emotions is abstract and high dimensional (Skerry and Saxe, 2015). Moreover, RSA could provide a novel method to investigate the representational structure down to the level of individual perspective rather than broad categorical information.

Although the representation of the human emotional expressions has been examined in many studies, it is not clear how the brain forms abstract emotional representations from whole-person’s stimuli and whether specific brain regions could show emotion-specific but stimulus category-independent (body, face, and whole-person) representations. Hence, in this study, we hypothesized that the emotion category was independent of three different perceptual cues (body, face, and whole-person) and could be decoded based on the activity patterns from different emotions. Functional magnetic resonance imaging (fMRI) data were collected when participants classified emotions (angry, fearful, and happy) expressed by videos of faces, bodies and whole-persons. First, we conducted the RSA to examine whether some specific brain regions contained emotion-specific but stimulus category-independent representations of perceived emotions. One possible abstract representation of emotions is that the neural patterns are significantly correlated in within-emotion conditions across stimulus types but uncorrelated in between-emotion conditions. To test this hypothesis, the dissimilarity matrix was first established for the abstract emotion model and then the searchlight-based RSA was performed to calculate the correlations between the dissimilarity matrix capturing the model and the neural dissimilarity matrix of each voxel across the whole brain. Then the cross-modal MVPA was performed as the additional validation analysis on the data to verify whether the clusters identified by whole-brain RSA were truly informative to abstract representations of emotions. A further univariate analysis was finally conducted to explore the differences between the mean activation patterns of the significant clusters in different conditions.

## Materials and Methods

### Participants

Twenty-four healthy volunteers were recruited in this study. All participants were right-handed, with normal or corrected-to-normal vision, and all declared having no history of neurological or psychiatric disorders. Four participants were excluded due to movement artifacts and twenty participants were finally included in the further analysis (10 females, mean age 21.8 ± 1.83 years, range from 19 to 25 years). This study was carried out in accordance with the recommendations of Institutional Review Board (IRB) of Tianjin Key Laboratory of Cognitive Computing and Application, Tianjin University with written informed consent from all subjects. All subjects gave written informed consent in accordance with the Declaration of Helsinki. The protocol was approved by the IRB of Tianjin Key Laboratory of Cognitive Computing and Application, Tianjin University. A separate group of volunteers (*n* = 18) from the same community participated in a preliminary behavioral experiment to evaluate stimuli delivering emotional contents most effectively.

### Experiment Stimuli

**FIGURE 1:**
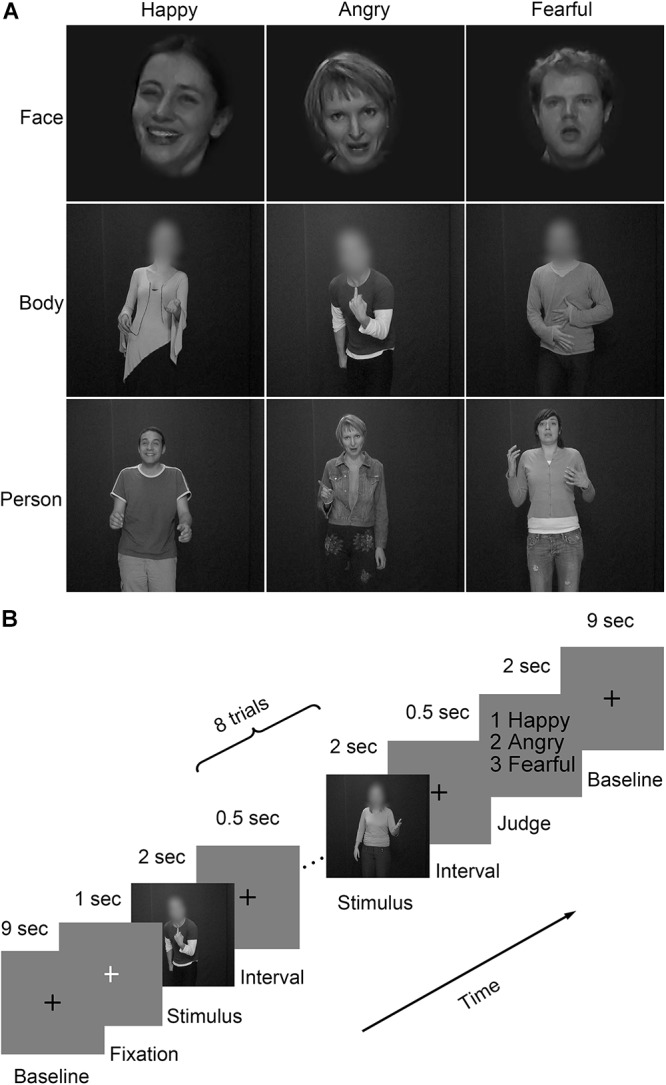
Representative stimuli and paradigm of the experiment design. **(A)** Three stimulus types (face, body, and whole person) expressing three emotions (happy, angry, and fearful) were used in the experiment. The faces and bodies were masked with Gaussian blur masks; **(B)** A schematic overview of the presentation timing for emotion judgment task. Participants performed four fMRI runs, each starting and ending with a 10 s fixation baseline period. Emotions expressed by three stimulus types (face, body, and whole-person) were presented in each of the first three runs. The last run, only used emotions expressed by the whole-person. Within each run, 18 blocks of eight trials of the same category were pseudo-randomly presented. These blocks were separated by 10 s fixation interval (a black cross was presented for 9 s, followed by a white cross presented for 1 s to control subjects’ attention). Each trial consisted of a 2 s video, followed by a 0.5 s interval. At the end of each block, participants were asked to make a choice between three emotions using a button press within a 2 s response window.

Video clips with three emotions (happiness, anger and fear) (Grezes et al., 2007; de Gelder et al., 2012, de Gelder et al., 2015) were chosen from the GEMEP (GEneva Multimodal Emotion Portrayals) corpus (Banziger et al., 2012). Twenty-four video clips (four male and four female actors expressed each emotion) were selected and processed in grayscale using MATLAB (Kaiser et al., 2014; Soria Bauser and Suchan, 2015). Video clips of emotional facial expressions and bodily expressions were cropped using Adobe Premiere Pro CC 2014 by cutting out and masking the irrelevant aspect with Gaussian blur masks (Kret et al., 2011), so that non-facial body parts were not visible in clips of emotional facial expressions and facial features and expressions were not visible in clips of emotional bodily expressions. Also, the face clips were magnified when necessary. All videos clips were trimmed or combined to exactly fit the duration of 2000 ms (25 frame/s) by editing longer- or shorter-length clips, respectively. The generated clips were finally resized to 720 pixel × 576 pixel and presented on the center of the screen. Representative stimuli for the main experiment were presented in **Figure 1A**.

The experiment included a total of seventy-two video clips (3 emotions × 3 stimulus types × 8 videos per condition). In advance of the current study, a behavioral experiment had been conducted with another group of participants (8 females, mean age: 21.9 years; 10 males, mean age: 22.4 years) without any known difficulties in emotional processing for stimulus validation. Raters were asked to categorize the emotional materials with six labels (anger, surprise, happiness, sadness, fear, and disgust) and rated the perceived emotion intensity at a 9-point scale. For each condition, expressions were well recognized (happy whole-person: 95%, angry whole-person: 95%, fearful whole-person: 87%, happy face: 97%, angry face: 86%, fearful face: 74%, happy body: 75%, angry body: 93%, fearful body: 82%). There were no significant differences in the intensity rates between the selected videos for three emotional expressions [happiness versus anger: *t*(17) = 0.73, *p* = 0.465; happiness versus fear: *t*(17) = 0.26, *p* = 0.796; anger versus fear: *t*(17) = 1.07, *p* = 0.285].

In order to examine the quantitative differences in the amount of movement between videos, the movement per video was assessed by quantifying the variation for each pixel in the intensity of light (luminance) between two adjacent frames (Grezes et al., 2007; Peelen and Downing, 2007). For each frame, we averaged the score (on a scale reaching a maximum of 255) higher than 10 (10 corresponds to the noise level of the camera) across the pixels to estimate movements. Then, these scores were averaged for each video. No significant differences were observed between all three emotional expressions [happiness versus fear: *t*(23) = 1.639, *p* = 0.108; happiness versus anger: *t*(23) = 0.833, *p* = 0.409; anger versus fear: *t*(23) = 2.045, *p* = 0.091].

### Procedure

The procedure consisted of four runs (**Figure 1B**), each starting and ending with a 10 s fixation. Three emotions (happiness, anger, and fear) expressed by three stimulus types (face, body, and whole-person) were presented in each of the first three runs. In the fourth run, only three kinds of the whole-person emotions (happiness, anger, and fear) expressed by the whole-person were presented. Within each run, eighteen blocks with eight trials were pseudo-randomly presented. These blocks were separated by a 10 s fixation interval (a black cross presented for 9 s and a white cross presented for 1 s to control subjects’ attentions). Each trial consisted of a 2 s video, followed by a 0.5 s inter-stimulus interval (ISI). At the end of each block, participants were asked to make a choice between three emotions using a button press within a 2 s response window. One block-designed localizer run was also performed, in which the stimuli included 4 types of static or dynamic faces, bodies, whole-persons, and objects. This run contains a total of 16 blocks (4 types × dynamic/static × repeat 2 times), and these blocks including 8 trials (1.5 s each) were separated by 10 s fixation interval. Each trial consisted of a 1.4 s stimulus, followed by a 0.1 s ISI.

### Data Acquisition

Functional images were acquired using a 3.0 T Siemens scanner in Yantai Hospital Affiliated to Binzhou Medical University with a twenty-channel head coil. Foam pads and earplugs were used to reduce the head motion and scanner noise (Liang et al., 2017). For functional scans, an echo-planar imaging (EPI) sequence was used (T2^∗^ weighted, gradient echo sequence), with the following parameters: TR (repetition time) = 2000 ms, TE (echo time) = 30 ms, voxel size = 3.1 mm × 3.1 mm × 4.0 mm, matrix size = 64 × 64, 33 axial slices, 0.6 mm slices gap, FA = 90°. In addition, a high-resolution anatomical image was acquired using a three-dimensional magnetization-prepared rapid-acquisition gradient echo (3D MPRAGE) sequence (T1-weighted sequence), with the following parameters: TR = 1900 ms, TE = 2.52 ms, TI = 1100 ms, voxel size = 1 mm × 1 mm × 1 mm, matrix size = 256 × 256, FA = 9°. The stimuli were displayed by high-resolution stereo 3D glasses of VisualStim Digital MRI Compatible fMRI system.

### Data Analysis

#### Behavioral Measures

For each participant, the response time and recognition accuracy of three kinds of emotions by three kinds of stimulus types were calculated. Then an analysis of variance (ANOVA) was performed on the accuracies to test the main effect and interactions between the factors Emotion and stimulus Category. Paired *t*-tests were further performed to examine the differences between all three emotions. The statistical analysis was performed using the SPSS 18 software.

#### Data Preprocessing

**FIGURE 2:**
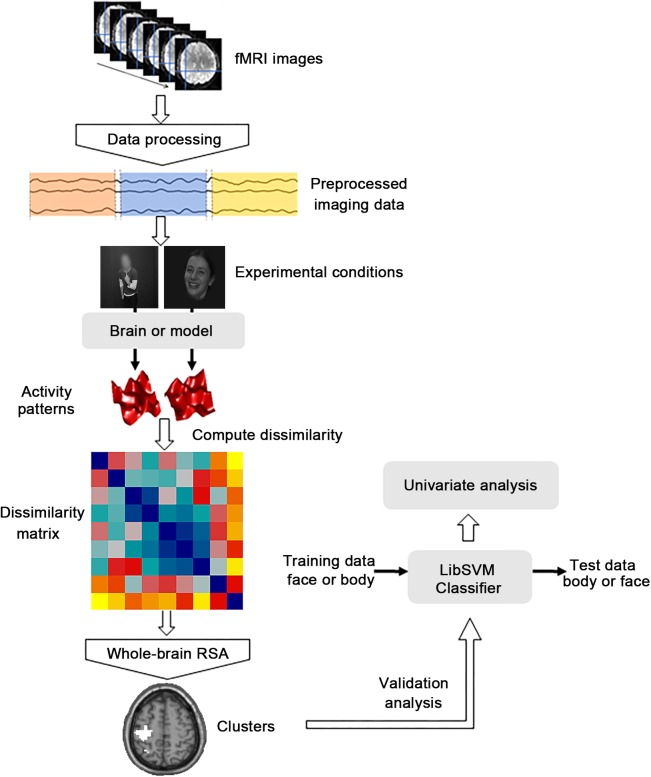
Flow chart of the main analytical steps. There were three kinds of main procedures. The dissimilarity matrix was first established for the abstract emotion model and then the representational similarity analysis (RSA) was performed to calculate the correlations between the dissimilarity matrix capturing the model and the neural dissimilarity matrix of each voxel across the whole brain. Then the cross-modal multivoxel pattern analysis (MVPA) was performed as the additional validation analysis to verify whether the clusters identified by whole-brain RSA were truly informative to abstract representations of emotions. A further univariate analysis was then conducted to explore the differences between the mean activation patterns of the significant clusters identified from the MVPA procedure.

Functional images were preprocessed and analyzed using the SPM8 software package^1^ and MATLAB software (The Math Works). The first five volumes corresponding to the baseline of each run for all functional data were discarded to allow for equilibration effects. Slice-timing corrected and spatially realigned to the first volume for head-motion correction were performed for the remaining 283 volumes. Subsequently, the T1-weighted images were segmented into the gray matter, white matter and cerebrospinal fluid (CSF) for normalization after being co-registered to the mean functional images. Then the generated parameters were used to spatially normalize the functional images into the standard Montreal Neurological Institute (MNI) space at an isotropic voxel size of 3 mm × 3 mm × 3 mm. Especially, the images in the first four runs and the functional localization run were smoothed with a 4-mm full-width at half-maximum (FWHM) Gaussian filter (Zhang et al., 2016; Liang et al., 2018). Before the further analysis, fMRI data were fitted with a GLM to obtain regressors for all nine experimental conditions (happy face, angry face, fearful face, happy body, angry body, fearful body, happy whole-person, angry whole-person, and fearful whole-person). The GLM was constructed to model the data for each participant and the subsequent analysis was conducted on each of the first three runs, generating nine activation patterns in total. The sources of nuisance regressors along with their time derivatives were removed through the linear regression, including six head motion correction parameters, and averaged signals from the white matter and CSF (Xu et al., 2017; Geng et al., 2018). The main analytical steps included in this study were shown in **Figure 2**. In the searchlight analysis and cluster-based MVPA, only the data of the first three run were used.

#### Representational Similarity Analysis

**FIGURE 3:**
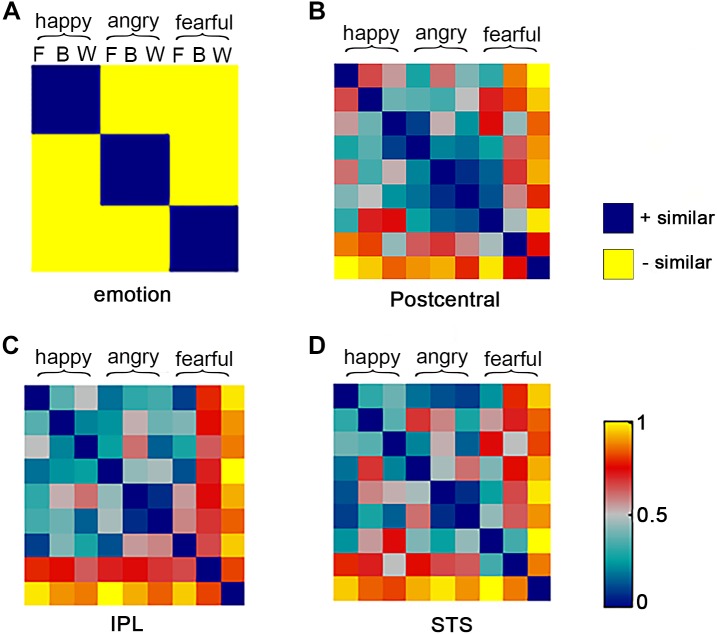
Representational similarity analysis model and neural representational dissimilarity matrices (RDMs). For one abstract emotion model **(A)** and three neural representation of three clusters **(B–D)**, the representational dissimilarity matrices were calculated. The model is indexed at two levels in the order in which experimental conditions are rearranged: emotion (happy, angry, and fearful) and stimulus category (F: face B: body W: whole-person). The model dissimilarity matrix illustrates hypothetical correlations of different activity pattern, for example, significant similarity between pair of conditions that from the same emotion (happy–happy pair or angry–angry pair or fearful–fearful pair) should be observed in the abstract emotion model. The neural RDMs illustrate the actual representation of these three brain areas.

To localize regions that supported abstract representation of three emotions generalize across three stimulus types, the whole-brain RSA was performed, in which the RSA framework for the whole brain “searchlight” analysis was constructed using the RSA toolbox (Nili et al., 2014). To estimate the neural representational structure of the brain, a dissimilarity matrix was established for the abstract emotion model as follows: the model was established by setting 0 for all conditions of within-emotion across stimulus types (i.e., happy face-happy body pair or angry face-angry whole-person pair) and 1 anywhere else. In other words, setting 1 for all conditions of between-emotion (i.e., happy face-angry face pair or happy face-angry body pair), as shown in **Figure 3A**. Considering that the diagonal entries were not relevant to the hypothesis, they were set as NaNs for the model and excluded from the following analysis.

In whole-brain searchlight analysis, a spherical volume-based searchlight approach (Kriegeskorte et al., 2006) was used. For each participant and each voxel in the brain, we selected a searchlight of 9-mm radius and established the neural dissimilarity matrix for this sphere with the following procedures: for each of the nine stimulus conditions (3 emotions × 3 stimulus types), the neural activity patterns estimated by GLM were extracted from this sphere. Pair-wise dissimilarities were then computed between each of two different activity patterns using the correlation distance (1 minus the Pearson correlation) based on the pattern of GLM weights across conditions, resulting in a symmetrical 9 × 9 RDM for each participant. To examine whether the representations were independent of stimulus types and to what extent the abstract emotion model could account for neural pattern information, we then computed the Kendall’s rank correlation coefficient (tau a) between the searchlight sphere and the abstract emotion model using the values derived from the upper triangles of neural RDMs and RDM of abstract emotion model. After that, the generated coefficients were assigned to the center voxel of the sphere in each searchlight analysis. This computational procedure was repeated across the whole brain for each individual, generating a whole-brain correlation map (r-map) for each participant (Nili et al., 2014). The group analysis was conducted based on the statistical r-maps, treating subjects as a random effect. One-side Wilcoxon sign-rank analysis was used to test in which voxel the correlation between the observed neural RDM and predicted model was significant by thresholding at *p* < 0.01 with a minimum cluster-size of 30 (resampled) contiguous voxels.

#### Cluster-Based MVPA

In the cluster-based analysis, the cross-modal MVPA was performed as an additional validation analysis to verify whether the clusters identified by the searchlight analysis were truly informative to abstract representations of emotions, where the validation analysis is necessary because the identified significant searchlight clusters are not guaranteed to be informative (Etzel et al., 2013). There were two main functions in our verification analysis. First, Type I error was prevented which might falsely infer the existence of modal-generic voxels that is not existent by requiring this analysis to confirm the effect. Second, like the *posthoc* testing of a common effect, the specific nature of the representation of emotions was tested to better describe these effects. Crucially, this analysis did not introduce any new effects, but could rather clarify the nature of the observed effects and serve as a conservative criterion for identifying these effects. The MVPA method used in the current study is similar to those methods that have been successfully used in the previous exploration of affective space (Baucom et al., 2012; Shinkareva et al., 2014; Kim et al., 2017). A logical regression classifier was used for the cross-modal classification to examine the abstract representation of emotions at the group level (Bishop, 2006). In details, the data of the three stimulus types (face, body, and whole-person) were extracted separately, each of which contained the data of three emotional types. Then the logistic classifier was trained from the data of one stimulus type (i.e., face), and then the data of the other stimulus type (i.e., body or whole-person) was used as a test set. The logistic classifier was trained/tested separately for each cluster. We made a two-way classification analysis (happy versus angry/fearful, which could be considered as positive versus negative: P vs. N) and a three-way classification (happy versus angry versus fearful: H vs. A vs. F) analysis, which decomposed the data in an orthogonal manner. Classification accuracies were averaged across two cross-validation folds (i.e., face to body and vice versa) for each participant. For each participant, the significant cross-modal classification provides strong evidence of the structural validity of the classification, as the classification is unlikely to be driven by associated variables, such as lower-level features (e.g., motion, brightness, hue, etc.) between different stimulus types. For the classification analysis between three emotional conditions, the one-sample *t*-test analysis was conducted to assess whether the group mean accuracy was significantly higher than the chance level (0.33). For the P vs. N classification analysis, half of the data for anger and fear conditions declined randomly, with each cross-validation equal to the baseline and therefore the chance level was 0.5. A one-sample *t*-test was also used to examine the significance of the group mean accuracy (the chance level was 0.5).

#### Analysis of the Differences Between Mean Activation Patterns

A further univariate analysis was conducted to explore the differences between the mean activation patterns of the significant clusters identified from the MVPA procedure in different conditions. For each cluster, the beta values were averaged across voxels for each condition as described in previous studies (Peelen et al., 2010; Kim et al., 2015). The generated mean activation values for each cluster were input into a 3 emotions × 3 stimulus types ANOVA. To test whether there were significant differences between the emotion-specific activations across stimulus types, we examined whether the mean activations estimated by the beta values in these clusters were more similar for within-emotion response than between-emotion response. To this end, for each participant, the mean response magnitudes in three stimulus types (faces, bodies, and whole-persons) were subtracted from the data. Subsequently, we compared the absolute differences between the same emotion across different stimulus types (e.g., happy faces vs. happy bodies) and the absolute differences between different emotions across different stimulus types (e.g., happy faces vs. fearful bodies).

## Results

### Behavior Analysis

The recognition accuracies of facial, bodily, and whole-person’s emotions were at a relatively high level (mean accuracy = 98.0%, SD = 5.3) (happy faces: 100%, angry faces: 97.5%, fearful faces: 96.7%, happy bodies: 97.5%, angry bodies: 97.5%, fearful bodies: 96.7%, happy whole-persons: 100%, angry whole-persons: 98.8%, and fearful whole-persons: 97.1%). The 3 × 3 ANOVA was performed on the accuracies with the factors Emotion (happy, angry, and fearful) and stimulus Category (face, body, and whole-person), without significant main effect for Emotion [*F*(2,38) = 2.98, *p* = 0.063] and stimulus Category [*F*(2,38) = 1.03, *p* = 0.367], nor any significant interaction effect between these factors [*F*(4,76) = 0.69, *p* = 0.599]. The 3 × 3 ANOVA of the response time with the factors Emotion (happy, angry, and fearful) and stimulus Category (face, body, and whole-person) showed no significant main effect for stimulus Category [*F*(2,38) = 1.91, *p* = 0.162] but for Emotion [*F*(2,38) = 20.53, *p* < 0.001], nor any significant interaction between these factors was observed [*F*(4,76) = 1.91, *p* = 0.118]. Additionally, we performed paired comparisons among three emotions irrespective of the stimulus category. The results showed that the response time of the subjects to happy emotions was shorter than that to angry emotions [*t*(19) = 3.98, *p* = 0.001] or fearful emotions [*t*(19) = 6.15, *p* < 0.001]. In addition, they responded to angry emotions significantly faster than fearful ones [*t*(19) = 2.75, *p* = 0.013]. **Table 1** showed the statistical details of the group-level behavioral data. In the emotion identification task, the recognition accuracies and response times for the nine conditions of the subjects were shown in **Table 1**.

**Table 1:** Mean emotion identification accuracies and corresponding response times.

Emotion	Category	Recognition rate (%)		Response time (ms)	
		
		Mean	SD	Mean	SD
Happy	Face	100	0	713.74	163.39
	Body	97.50	6.11	669.36	160.87
	Whole-person	100	0	675.25	155.35
Angry	Face	97.50	6.11	808.22	235.30
	Body	97.50	6.11	762.83	227.69
	Whole-person	98.75	3.05	767.05	224.22
Fearful	Face	96.67	6.84	809.54	234.73
	Body	96.67	6.84	825.16	220.20
	Whole-person	97.08	5.59	836.10	210.54


### Searchlight Similarity Analysis

**FIGURE 4:**
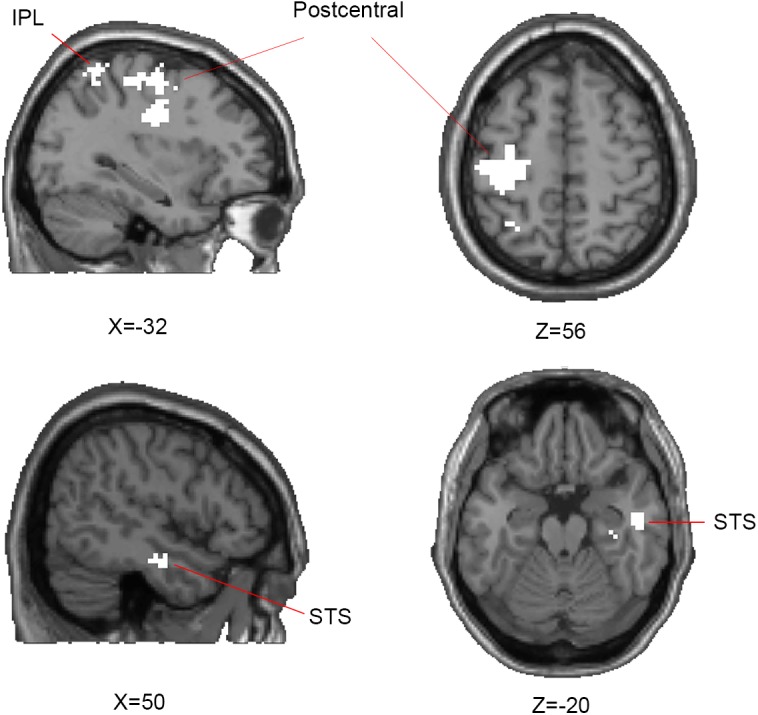
Results of representational similarity analysis of whole-brain searchlight showing the significant clusters of abstract representation of emotion. For abstract representation of three emotions, three clusters are significantly correlated with the abstract emotion model: the left postcentral gyrus, inferior parietal lobe (IPL), and right superior temporal sulcus (STS) which are shown on axial and sagittal slices. One-side Wilcoxon sign-rank test was used in the second-level group analysis and p-map generated by thresholding at *p* < 0.01 with a minimum cluster-size of 30 contiguous voxels.

In the searchlight similarity analysis, we compared the neural RDMs with the hypothetical abstract emotion model across the whole brain. The abstract emotion model was established by setting 0 for all conditions of within-emotion across stimulus types and 1 for all conditions of between-emotion, as shown in **Figure 3A**. The model RDM were indexed at two levels in the order where experimental conditions were rearranged: emotion (happy, angry, and fearful) and stimulus category (face, body, and whole-person). The model dissimilarity matrix illustrated the hypothetical correlations of different activity patterns for each condition, for example, the significant similarity between pair of conditions that from the same emotion (happy–happy pair or angry–angry pair or fearful–fearful pair) was observed in the abstract emotion model. Then the correlation map (r-map) for each participant was obtained and the random-effect group analysis (*N* = 20) was performed on the individual correlation map, revealing that all three clusters were significantly correlated with the abstract emotion model (*p* < 0.01, cluster size >30): left postcentral gyrus (-39, -21, 36), left IPL (-30, -54, 54), and right STS (51, -9, -24), as shown in **Figure 4**. The neural RDMs of the three clusters illustrated the actual representation of these brain areas. The neural RDMs, like the model, were also indexed at two levels) (**Figures 3B–D**). We discovered that the RDMs of specific brain regions were similar to the model to a certain extent. The correlations between within-emotions conditions were relatively high. For example, the correlations between three types of stimuli for the within-angry emotion conditions were relatively higher than the between-emotion conditions. The whole-brain searchlight analysis revealed that the abstract emotion model was positively related to the neural similarity in the left postcentral gyrus, left IPL, and right STS. The significantly positive correlations between the model and neural RDMs suggested an abstract representation of emotions in these three regions. **Table 2** depicted the detailed MNI coordinates of these clusters.

**Table 2:** Significant clusters (*p* < 0.01, cluster size >30) correlated with the abstract emotion model from searchlight analysis.

Anatomical region	Hemisphere	Cluster size	MNI coordinates			peak
			*x*	*y*	*z*	intensity
Postcentral gyrus	L	323	-39	-21	36	28.99
STS	R	48	51	-9	-24	15.61
IPL	L	34	-30	-54	54	17.65


*The cluster size indicates number of voxels; STS, superior temporal sulcus; IPL, inferior parietal lobe; R, right; L, left*.

### Cluster-Based Cross-Modal MVPA

Cluster-based MVPA was conducted as a validation analysis to test whether clusters identified by the searchlight analysis contained information on the type of emotions, as proposed by Etzel et al.’s (2013) “Confirmation Test.” The clearest evidence for an abstract representation of emotions is that the searchlight similarity analysis and MVPA converged on the same result, in other words, the clusters selected by the searchlight analysis could successfully distinguish either P vs. N or H vs. A vs. F for the cross-modal classification. Therefore, we only included those clusters that could distinguish either P vs. N or H vs. A vs. F using MVPA for any two or three of these three stimulus type pairs (face-body, face-whole person, and body-whole person). The clusters, which could not distinguish both P vs. N and H vs. A vs. F for all three pairs, were excluded from the further analysis. Classifying angry and fearful stimuli together versus happy stimuli (P vs. N) is important because the variation in this dimension clearly distinguishes bivalent representations and some brain regions may not be fine-grained to classify the three emotions, but can sort out different valences.

The cross-modal classification accuracies of three types of emotions for the three stimulus type pairs were higher than the baseline (0.33) for the left postcentral gyrus (face-body pair: 38.18, face-whole person pair: 34.29, body-whole person pair: 36.42) but lower for the STS and IPL. Similarly, the classification accuracies of P vs. N for three stimulus type pairs were higher than the chance level (0.5) for the left postcentral gyrus (face-body pair: 55.35, face-whole person pair: 52.92, body-whole person pair: 55.52) but not for the STS and IPL. Details were shown in **Table 3**. Additionally, one-sample *t*-tests in the left postcentral gyrus found that the classification accuracies for the face-body pair [*t*(19) = 3.70, *p* = 0.002] and body-whole person pair [*t*(19) = 4.15, *p* = 0.001] were significantly higher than 0.33 as distinguishing three types of emotions, and significantly higher than 0.5 as distinguishing P vs. N for the face-body pair [*t*(19) = 4.14, *p* = 0.001], face-whole person pair [*t*(19) = 2.16, *p* = 0.044] and body-whole person pair [*t*(19) = 5.83, *p* < 0.001], indicating that the left postcentral gyrus was informative on the type of emotions of all three stimulus types (face, body, and whole-person). The remaining two clusters could not distinguish the P vs. N or H vs. A vs. F significantly for any of the three pairs (*p* > 0.05).

**Table 3:** Classification accuracies from cross-modal MVPA for each searchlight cluster.

Clusters	Hemisphere	H vs. A vs. F			P vs. N		
		F-B	F-W	B-W	F-B	F-W	B-W
Postcentral gyrus	L	38.18^∗^	34.29	36.42^∗^	55.35^∗^	52.92^∗^	55.52^∗^
STS	R	33.04	31.17	32.85	52.02	50.46	49.15
IPL	L	33.06	31.29	32.21	51.56	48.25	48.69


*The cluster size indicates number of voxels; STS, superior temporal sulcus; IPL, inferior parietal lobe; ^∗^p < 0.05; R, right; L, left; H vs. A vs. F, happy versus angry versus fearful; P vs. N, happy versus angry/fearful which could be seen as positive versus negative; oxF-B, F-W, and B-W indicate the three stimulus type pairs (face-body, face-whole person, and body-whole person).*

#### Mean Activation in the Identified Clusters by the Searchlight Analysis

**FIGURE 5:**
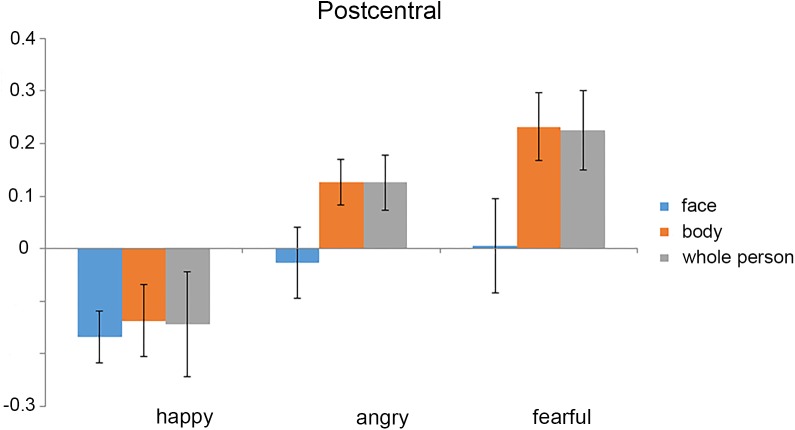
Mean activation (parameter estimates) for each experimental condition in the left postcentral gyrus. The mean activation was extracted from the left postcentral gyrus by averaging the stimulus-related activations [as estimated from the general linear models (GLMs)] across all the voxels. We found that the mean activations in all of the stimulus conditions were below the baseline. Error bars show SEM.

The mean activation was extracted from the left postcentral gyrus by averaging the stimulus-related activations (as estimated from the GLMs) across all the voxels (**Figure 5**). The 3 × 3 ANOVA for parameter estimates of mean activation with the factors stimulus Category (face, body, and whole-person) and Emotion (happiness, anger, and fear) showed a main effect for Emotion [*F*(2,38) = 7.34, *p* = 0.002] and a weak trend towards main effect for stimulus Category [*F*(2,38) = 3.07, *p* = 0.058], and a weak trend towards interaction between these factors [*F*(4,76) = 2.18, *p* = 0.079] in the left postcentral gyrus, suggesting that the activation differences between the emotions differed in all three modalities. To further confirm whether there were the emotion-specific activation differences across stimulus types, we tested whether the average activations estimated by the beta values in the left postcentral gyrus were more similar for the within-emotion response than between-emotion response. If the mean activation values carried emotion-specific information, the mean within-emotion response difference should be smaller than the mean between-emotion response difference. However, the results showed that the mean within-emotion response difference was equivalent to the mean between-emotion response difference for all three stimulus type pairs in the postcentral gyrus (*p* > 0.05, for all tests). Thus, this confirmed that the mean response amplitude values might not provide emotion-specific information across different stimulus types.

## Discussion

In this study, we used a searchlight-based RSA to investigate the regions that could represent emotions independent of the stimuli (body, face, and whole-person) mediating the emotions. The searchlight RSA with an abstract emotion model identified three clusters (left postcentral gyrus, left IPL, and right STS) that contained information about specific emotions regardless of the type of stimulus. Furthermore, the additional validation analysis found that the neural activity pattern in the left postcentral gyrus could successfully distinguish the happy versus angry/fearful conditions (positive versus negative, P vs. N) for all the three stimulus type pairs and three types of emotions (H vs. A vs. F) for two stimulus types pairs (body versus face and body versus whole-person), when the cross-modal classification analysis was performed. The other two clusters could not make a successful classification of the P vs. N or H vs. A vs. F for any of the three pairs. Therefore, the results further confirmed that the left postcentral gyrus played a crucial role in emotion representation at an abstract level. The univariate analysis showed that the mean within-emotion response difference was significantly smaller than that between-emotion response difference for all three stimulus type pairs in the left postcentral gyrus. This further confirmed that the left postcentral gyrus was truly informative of emotions of face, body and whole-person. Therefore, these findings provide evidence for emotion-specific representations in the left postcentral gyrus independent from the stimulus types (body, face, and whole-person) that conveyed the emotions.

### Representations of the Facial, Bodily, and Whole-Person’s Emotions

The main goal of this study was to explore the regions that could represent emotions at an abstract level. Using a searchlight-based RSA, the left postcentral gyrus was identified to be capable of distinguishing three types of emotions for face-body pair and body-whole person pair and distinguishing happy versus angry/fearful (like positive versus negative) for all the three pairs. The left postcentral gyrus has often been shown to be involved in emotion processing (Viinikainen et al., 2010; Baucom et al., 2012; Kassam et al., 2013; Sarkheil et al., 2013; Flaisch et al., 2015; Kragel and LaBar, 2015). And it also has been found to be involved in emotional face and body processing (van de Riet et al., 2009) and the recognition of vocal expressions. One latest study confirmed the successful decoding of the affective category from perceived cues (facial and vocal expressions) by the activation patterns in the left postcentral gyrus, which was consistent with our study (Kragel and LaBar, 2016). Another recent research identified that the postcentral gyrus was critical for valence decoding. Furthermore, in the postcentral gyrus, the information about valence (positive, neutral, and negative) was represented in activation patterns elicited by viewing photographs of different affective categories (Baucom et al., 2012), which was consistent with the present study. The postcentral gyrus also has been implicated in the perception of emotions based not just on facial expressions, but also on vocal prosody and whole-person’s expressions, when subjects made emotion judgments (Heberlein and Atkinson, 2009). Consistent with our study, it indicated that the role of the postcentral gyrus in emotion recognition extended to recognizing whole-person emotional expressions. A previous finding revealed an interaction effect between emotion type and intensity corresponding to level of aversiveness in the postcentral gyrus when decoding dynamic facial expressions (Sarkheil et al., 2013). In contrast, our study only focused on the change in the emotion type, keeping the intensity unchanged. But we still found the abstract representation of face, body and whole-person in postcentral gyrus when the emotions were classified, suggesting that the extended cortical networks might be involved in processing the highly integrated information of face, body or whole-person stimuli. The postcentral gyrus also has been shown to make a consistent contribution to the cross-condition classification of four basic emotions induced by the video clips and imagery through emotion words. It indicated that the neural signatures were consistent within different emotions from video and imagery and suggested that the abstract representation of emotions was independent of the emotion induction procedure within the postcentral gyrus (Saarimaki et al., 2016). Consistent with this interpretation, our current results provide further evidence for the role of the left postcentral gyrus in abstract emotional processing by encoding emotional information regardless of the stimuli types (face, body, and whole-person). Furthermore, one recent study examined the neural representations of the categorical valence (positive, neutral, and negative) elicited by visual and auditory stimulus modalities, suggesting evidence for modality-general representations in the left postcentral gyrus (Kim et al., 2017). This finding directly compared different valence states, but specific emotional categories were not considered. Our study provided further evidence for the role of the left postcentral gyrus in abstract emotional processing by encoding three emotions (happiness, anger, and fear) expressed by different stimulus types. The searchlight-based RSA revealed an emotion-specific but stimulus category-independent neural representation in the left postcentral gurus. Our study suggested that abstract representations of emotions could extend from the face and body stimuli to whole-person stimuli. In summary, the findings of the current study provide support for the left postcentral gyrus for abstract representations of emotions.

The cross-modal classification of three emotions found that the mean accuracy was not significant for the face-whole person pair in the left postcentral gyrus. We speculated that as most of the low-level features (e.g., motion, brightness, hue, etc.) could not be shared between these two stimulus types, the effect of low-level features on a logical regression classifier trained from one stimulus type (face or whole-person) might not play a role in the testing of the other stimulus types (whole-person or face). In this study, only the construct of visual emotions were represented, which was easier to be influenced during the classification of three emotions. As previously demonstrated, this is because the encoding of the visual affect might be highly affected by the lower-level features in the visual modality (e.g., motion, brightness, hue, etc.) (Gabrielsson and Lindström, 2001; Lakens et al., 2013). Therefore, the differences between the stimuli of different emotional categories may be due to the accompanying differences of lower-level features rather than the emotional differences, which led to the classification performance for the face-whole person pair was not significant. Another possibility is that the left postcentral gyrus may not be fine-grained to classify the three emotions, but can sort out different valences (Peelen et al., 2010; Baucom et al., 2012; Kim et al., 2017). In recent researches, the postcentral gyrus was proved to be critical for valence decoding (Baucom et al., 2012; Kim et al., 2017), but insensitive to specific emotion categories, which could not successfully decode the specific affective categories from perceived cues (facial, bodily and vocal expressions) when the cross-modal classification analysis was performed (Peelen et al., 2010). In our present study, the left postcentral gyrus could still distinguish three types of emotions using MVPA for the two stimulus type pairs (face-body and body-whole person) and P vs. N for all the three stimulus type pairs, indicating that the left postcentral gyrus was informative to emotion representation of all three stimulus types (face, body, and whole-person) (Heberlein and Atkinson, 2009; Kragel and LaBar, 2016; Kim et al., 2017).

### Regions Where Emotions Could Not Be Represented

The searchlight-based RSA revealed that the neural RDMs of the IPL and rSTS were significantly correlated with the abstract emotion model. But both clusters could not make a significant classification between all three kinds of emotions and P vs. N for any of the three stimulus type pairs, suggesting that the significant correlations between neural RDMs of the IPL and rSTS and the abstract emotion model possibly arose from the overpowered nature of the searchlight analysis. Because the Type I error which might falsely infer the existence of modal-generic voxels that is not existent when the searchlight analysis was performed (Kim et al., 2017). And it might cause us to conclude that the hypothetical effects or relationships exist but in fact it doesn’t. Therefore, our finding indicated that the IPL and rSTS might not contain enough information to make an abstract representation for facial, bodily, or whole-person’s feelings.

The IPL and rSTS have been demonstrated that facial and bodily emotions can be coded at an abstract level regardless of the sensory cue in previous studies (Peelen et al., 2010; Watson et al., 2014; Jessen and Kotz, 2015; Kim et al., 2017), but these studies have focused only on the abstract representations elicited from single face or body parts. Furthermore, our study confirmed the unsuccessful cross-modal decoding of the affective category from perceived cues (facial, bodily, and whole-person’s expressions) by the activation patterns in the IPL and rSTS, suggesting that the abstract representations of emotions might not extend from the face and body stimuli to whole-person stimuli in both clusters. However, the latest evidence for modality-general representations in the IPL and rSTS has been confirmed by examining the neural representations of the categorical valence (positive, neutral, and negative) by visual and auditory stimulus modalities. Similarly, the inferior parietal cluster didn’t show significant P vs. N (positive versus negative) or PN vs. 0 (positive/negative versus neutral) classification which was consistent with our study, but significant information from multidimensional scaling (MDS) (Kim et al., 2017) results for both P vs. N and PN vs. 0 representations. The confirmatory MDS analyses confirmed that this cluster is informative to valence representation, which might not be applicable to our results, because three kinds of visual stimuli were used in our experiment without auditory stimuli. Similarly, future work is needed to model the similarity structure dimensionally using a MDS method for our two clusters to reveal the underlying mechanisms when perceiving the emotions of face, body and whole-person, and to explore whether both clusters could represent all three stimulus types’ emotions at an abstract level.

Behavioral and neuroimaging studies have suggested that the human brain could encode whole-persons in a holistic rather than part-based manner (McKone et al., 2001; Maurer et al., 2002; Zhang et al., 2012; Soria Bauser and Suchan, 2013), indicating that the whole-person’s emotional expression might not be integrated from the isolated emotional faces and bodies. Our latest study has found that in the EBA, the whole-person patterns were almost equally associated with weighted sums of face and body patterns, using different weights for happy expressions but equal weights for angry and fearful ones (Yang et al., 2018), but this was not established for the other regions. Although some other regions like MPFC, left STS and precuneus have been demonstrated to be capable of coding the facial and bodily emotions at an abstract level regardless of the sensory cue (Peelen et al., 2010; Klasen et al., 2011; Chikazoe et al., 2014; Skerry and Saxe, 2014; Aube et al., 2015; Kim et al., 2017; Schirmer and Adolphs, 2017), these regions that were not sensitive to whole-person stimuli might not be able to represent the emotions of whole-person stimuli (Tsao et al., 2003; Pinsk et al., 2005; Heberlein and Atkinson, 2009). A recent study found that there existed a cross-modal adaptation in the right pSTS using dynamic face-to-voice stimuli, suggesting the presence of integrative, multisensory neurons in this area (Watson et al., 2014). Similar results were observed when body-to-voice stimuli were used (Jessen and Kotz, 2015). It indicated that the integration of different stimulus types might attribute to interleaved populations of unisensory neurons responding to face, body or voice or rather by multimodal neurons receiving input from different stimulus types. By examining the adaptation to facial, vocal and face-voice emotional stimuli, the multisensory STS showed equally adaptive responses to faces and voices, while the modality-specific cortices, such as face-sensitive and voice-sensitive cortices in STS, showed a stronger response to their respective preferred stimuli (Ethofer et al., 2013). Hence, the IPL and rSTS that have been demonstrated to be capable of representing facial and bodily emotions at an abstract level might not had the integrative, multisensory neurons which can adapt to whole-persons’ emotion. Therefore, the IPL and rSTS might not be able to represent the emotions extending beyond face and body stimuli to whole-person stimuli.

### Limitation

There are several limitations to be addressed in this study. The first one is that the emotional state was expressed only via the visual modality. Therefore, our results may not be generalized to other modalities, such as auditory or tactile emotional stimuli. The purpose of our study was to investigate whether there were specific regions that could represent whole-person’s emotions with facial and bodily emotion expressions abstractly. Three types of emotional stimuli of visual modality were used in our study. However, as there is no auditory or tactile condition, our research is limited to a certain extent. Future work is needed to explore the cross-modal representation of emotions. Another limitation is that the generality of our findings may be influenced by the relatively small sample size (*N* = 20) for functional MRI studies on healthy participants. To verify whether the number of participants was valid, the pre-determined sample size was computed with a priori power analysis which was conducted using the statistical software G^∗^Power^2^. The analysis showed that the sample size of this study was moderate and our results remained valid and efficient. Although many studies on the emotional expressions had comparative sample size (Peelen et al., 2010; Kim et al., 2015, 2016, 2017), a larger sample size could better prove the effectiveness of our findings and a bigger statistical power can be obtained. So, replicating this study with a larger number of participants appears considerable in the future work. In addition, examining the potential of age-related differences between different age groups is also an issue worthy of study in the future.

## Conclusion

We found emotions can be represented at an abstract level using three emotional stimuli in the left postcentral gyrus, left IPL and rSTS by using a whole-brain RSA. These three clusters probably contain emotion-specific but stimulus category-independent representations of perceived emotions (happy, angry, and fearful). Further cluster-based MVPA revealed that only the left postcentral gyrus could distinguish three types of emotions and happy versus angry/fearful which could be considered as positive versus negative for the two or three stimulus type pairs. Future research will be needed to model the similarity structure dimensionally using a MDS method for the IPL and right STS to reveal the underlying mechanisms when the face, body and whole-person expressions are perceived.

## Author Contributions

BL and XY designed the experiments. JX, XY, and XL performed the experiments. LC and JX analyzed the results. LC wrote the manuscript. JX, BL, and XL contributed to manuscript revision. All authors contributed to discuss the results and have approved the final manuscript.

## Conflict of Interest Statement

The authors declare that the research was conducted in the absence of any commercial or financial relationships that could be construed as a potential conflict of interest.

## Footnotes

1 http://www.fil.ion.ucl.ac.uk/spm/software/spm8/

2 http://www.gpower.hhu.de/
